# Supplementary material for: OpenMM 7: Rapid development of high performance algorithms for molecular dynamics
Source: PLoS Comput Biol. 2017 Jul 26;13(7):e1005659. doi: 10.1371/journal.pcbi.1005659 (PMC5549999; doi:10.1371/journal.pcbi.1005659)
Supplement: S1 Source Code — (ZIP) [file pcbi.1005659.s002.zip › OpenMM-7.0.1-Source/docs/api-python/generated/simtk.openmm.app.topology.Chain.html]

Chain — OpenMM 7.0.1.dev-5e86c4f documentation


# Chain¶

*class* `simtk.openmm.app.topology.``Chain`(*index*, *topology*, *id*)¶
:   A Chain object represents a chain within a Topology.

    `__init__`(*index*, *topology*, *id*)¶
    :   Construct a new Chain. You should call addChain() on the Topology instead of calling this directly.

    Methods

    |  |  |
    | --- | --- |
    | `__init__`(index, topology, id) | Construct a new Chain. |
    | `atoms`() | Iterate over all Atoms in the Chain. |
    | `residues`() | Iterate over all Residues in the Chain. |

    `residues`()¶
    :   Iterate over all Residues in the Chain.

    `atoms`()¶
    :   Iterate over all Atoms in the Chain.

    `__delattr__`¶
    :   x.\_\_delattr\_\_(‘name’) <==> del x.name

    `__format__`()¶
    :   default object formatter

    `__getattribute__`¶
    :   x.\_\_getattribute\_\_(‘name’) <==> x.name

    `__hash__`¶

    `__reduce__`()¶
    :   helper for pickle

    `__reduce_ex__`()¶
    :   helper for pickle

    `__setattr__`¶
    :   x.\_\_setattr\_\_(‘name’, value) <==> x.name = value

    `__sizeof__`() → int¶
    :   size of object in memory, in bytes

    `__str__`¶

High performance molecular simulation on GPUs


### Navigation

- Application Layer
  - Loaders and Setup
  - Representation and Manipulation
  - Simulation
  - Reporting Output
  - Extras
- Library Layer

©2015, Stanford University and the Authors.
|
Powered by Sphinx 1.3.1
& Alabaster 0.7.7
|
Page source
